# A Plea for Hospital Delivery of Our Maternity Patients1Read before Section of Obstetrics and Gynecology, Buffalo Academy of Medicine, April 26, 1910, and published in the Buffalo Medical Journal by resolution of the Section

**Published:** 1911-05

**Authors:** Ludwik Schroeter

**Affiliations:** Buffalo, N. Y.


### A Plea for Hospital Delivery of Our Maternity Patients[1]

By LUDWIK SCHROETER, M.D., (Bern)
Buffalo, N. Y.

Attending Obstetrician, St. Mary's Maternity Hospital
Assistant Attending Obstetrician, Buffalo General Hospital
Clinical Instructor in Obstetrics, Medical Department, University of Buffalo

IT is a well established fact that superstition and prejudice are legitimate offspring of ignorance. The less one knows about a subject the greater the chances that he will develop superstitious opinions about it. The less one knows of a person, class of people, a nation or an institution, the more apt he is to be prejudiced against them. This truism is most applicable in the domain of medicine. Here ignorance on one hand, and the strongest of instincts—self preservation on the other—develop and foster these two pathological phenomena of the human intellect. If we analyse the superstitions adhered to by the public in medical matters, we find that the bulk of them is nothing more than standard theories of the medical profession of decades or centuries gone by. In all probability our own mistaken ideas of today will be culture media for the layman's superstitions of future generations. We may laugh and scoff at the dread many of our patients have of fresh air but it would suffice to open a textbook of fifty years ago to find similar ideas of "Rheumas" etc. The prominence placed by most mothers on the teething process of their children between the ages of six months and three years can be explained by referring to authorities of the past generation. Nay! Even as late as ten years ago, a highly intelligent and prominent member of our profession expressed himself that he considers the gum-lance so important an instrument that, if compelled to have his choice, he would rather discard all other surgical appliances than this not valuable one. The pet theory of the classical grape seed or cherry pit as an etiological factor in appendicitis is too fresh in our memories to remind you of it. Many young men, however, who enter our profession today, will soon shake their heads, wondering at their simple minded patients who may entertain such a fantastic notion. To mention the host of superstitions which reign undisputed in the minds of the practical obstetrical nurses and their patients would fill my paper to its brim; yet a few striking examples may not be amiss: Why should not a naive young mother believe the explanation of her granny that the birthmark on the body of her little darling is due to the sight of fire; a superfluous finger

1. Read before Section of Obstetrics and Gynecology, Buffalo Academy of Medicine, April 26, 1910, and published in the BUFFALO MEDICAL JOURNAL by resolution of the Section to the fright, caused by a maimed beggar, who vividly scared her imagination just a month before her delivery? We find elaborate essays, discussing the possibility, nay—even the probability of maternal impressions, in reputable medical journals of today.

While a member of the Board of Examiners in Midwifery, I was puzzled during many years by one mistake, repeated by the majority of applicants. When asked "What may be the danger to the child if the umbilical cord prolapses?" most of them would answer promptly and invariably that the danger consists in chilling of the cord. I could not believe that this international harmony, that this uniformity of a mistaken notion running through so many years and coming from applicants of so many different countries should be only a coincidence. Recently I found an explanation. I came across the same opinion advocated in an old textbook of obstetrics by men of such eminence in their time as Velpeau, Guillenot and others. They claimed that "When the cord prolapses beyond the vulva, the blood may lose its fluidity in consequence of being chilled by the external temperature, perhaps may even coagulate." So great has the influence of the medical profession been for centuries and so strongly do physicians impress even today their opinions upon the minds of the public, that they are directly and indirectly responsible, not only for wrong notions and superstitions, but also for their prejudices in medical matters. The public opinion of today, relative to hospitals is nothing more than the distorted reflection of the views maintained by the medical profession of the past generation. It certainly would be interesting reading to follow up the views held by physicians in different periods regarding hospitals. At their very inception, hospitals had an altogether different character than that of today; as the name indicates they served rather as places of refuge and safety from persecution; gradually they assumed a religious character with an admixture of what is now denominated "Social Service." This characteristic hospitals retained for many centuries.

During the latter half of the nineteenth century by a process of evolution more and more of the hospitals became secular institutions. This process of emancipation of hospitals is progressing so rapidly that the future generations of physicians will be puzzled at the names of the older institutions, such as "Hotel Dieu," "Hospital St. Lazare," "St. Luke's Hospital," etc. This metamorphosis of the character of hospitals had an enormous influence upon the standing of physicians. While in former years our profession used to be considered, and considered itself only as an appendix of the institution, in later years, especially after the tremendous progress in surgery and the development of laboratory and research work, the situation changed radically. As late as a few decades ago hospitals used to be only charitable institutions under the control of ecclesiastics, harboring mostly indigent poor and the dregs of society, sometimes connected with medical colleges, nowadays catering to all classes of society. Some hospitals are genuine palaces with apartments equipped sometimes so lavishly that they look more like bridal chambers than sick rooms. Such a great change in the intrinsic character of hospitals must have had its important causes.

The experience of surgeons during the last twenty-five years or so proved to them beyond peradventure the fact that not only is the mortality greatly reduced in patients operated in properly arranged hospitals as compared with identical cases operated in patients' houses, even under the most favorable circumstances, but that after operation complications happen much more infrequently in institutions and that the duration of the after treatment is much shorter there. This discovery made the more prominent surgeons hesitate at first, and later to flatly refuse to operate in private homes. This radical stand of the elite of the profession compelled the rest of the surgeons to follow suit, and it did not take many years before the public at large thoroughly understood the advantages of hospital treatment of surgical cases and today we do not hear any more of the "prejudice" of the public—nay! it is sometimes even hard to restrain a patient with a minor surgical affliction from hastening to a hospital.

Gynecologists did not wait long before they followed the sensible example of their senior professional brethren. Quite in their foot-steps we see the great phalanx of general practitioners follow. They also came to the conclusion that a case of typhoid fever, pneumonia, or acute encarditis for instance, has better chances of recovery in a hospital than at home; especially since in later years the institution of trained nurses has become more and more popular. Therefore it strikes us particularly when we find an article in the Encyclopedia Brittanica saying "Although hospitals have been intended as a blessing and a benefit to the poor, they have too often proved the reverse. So much was this the case formerly that it has been not infrequently debated whether hospitals are or are not gigantic evils, and even where it is admitted that they are of value in cases of actual disease, it is still doubtful if they are really of benefit in cases of confinements." The first part of this quotation hardly requires discussion. The general view of the profession radically differs from it. The multiplication of hospitals during the last twenty-five years, but unheard of in former times, disproves this pessimistic view.

In Buffalo, for instance, during the last quarter of a century, the proportion of population to the number of hospitals and hospital beds shows a remarkable change. While in the year 1885 there existed in our city six hospitals with 335 beds to a population of nearly 255,000; in 1910, with a population of 423,000, we have nineteen hospitals with 1,700 beds, or with an increase of population by 1.62, the increase of hospitals and beds is 5.2 or more than three times as much.

The purpose of my paper is to prove that what has been of so great a benefit to surgical, gynecological, and the internal diseases applies with equal force to obstetrical cases.

Whenever the subject is discussed even today, we find quotations from statistics of a hundred years ago which showed that while the mortality of women delivered in hospitals was thirty-five per mille, those delivered in their homes showed only a fraction over four per mille. We know that at present just the opposite is the fact, that the mortality of women in labor is about eight times higher in private practice than in modern maternities.

This opinion is held by nearly all authorities in obstetrics and is based on accurate statistics, both hospital and municipal, as kept in most of the cultured European countries.

The fact that the lives of about twenty thousand mothers could be saved each year in the United States, if the post partum mortality could be brought to the hospital norme, ought to be sufficient argument, I think, in favor of my proposition. If more than seventy mothers could be saved in our city each and every year, it is certainly worth while to spend some time in discussing this problem. But whenever this subject is touched—the same mistake is being made—we are considering in our arguments only the lives of mothers—we forget the wholesale slaughter of the innocent, we forget that tens of thousands of children could be saved in the United States annually, if labors were conducted in an ideal manner in maternities. The ideal to be aimed at in obstetrical practice is, however, not merely a living mother and a living child, but rather a *living healthy mother* and a *living, healthy child.* How far we are from this ideal every one of you is well aware. Let those of you who doubt it pass through gynecological wards, read the histories of their inmates, let them study carefully the reports of nerve specialists, and let them scan the year-books of the institutions for the blind.

There is a class of physicians who object in principle to sending obstetrical cases to hospitals; some others agree to this proposition only in exceptional cases, in which a complicated operation is anticipated; lastly, there are many who agree with this plan, but bring out as an objection the prejudice of the public against maternities.

Let us first consider the last category. As I stated before, the public prejudice is nothing but an excuse for their ignorance, and in most cases more or less modified echo of opinions of the medical profession. If, therefore, the general practitioner can be convinced that it is in the best interest of their patients, if they are delivered in maternities, it will only be a question of a short time when the majority of women will be not only willing, but anxious to avail themselves of institutions which are the safest refuge to them and their progeny, where their imagination will not be haunted any longer by possible accidents of childbed, when many a woman will cease to think of infanticide as a self-defense to escape this imaginary terror.

To fully realise the condition of affairs we must first get rid of the notion that "labor is a physiological act." Imagine for a moment what would become of our race if every physiological act would have as a result a mortality considered at present as ideal in obstetrics, 1:1000. No! Labor is by no means a physiological function. At best it oscillates between physiology and pathology, with a strong inclination toward the latter. If we take into consideration as a factor—also, that in the new-born, vertex presentations constitute 97 per cent. of all the labors, there remains, then, to start with, 3 per cent. of the cases of potential abnormalities to mother, child, or both. But taking a case of vertex presentation, we are not insured against the possibility of a prolapse of the cord in the course of labor. A post-partum hemorrhage, if at all severe, is certainly a condition that requires intelligent assistance of trained minds and hands. It certainly is an accident which cannot be foreseen and is liable to happen in cases of the most robust women. Averting the consequences of such an accident alone would be enough reason to place a woman in the hospital, if she wants to take the least possible chances with her life, especially so if there should occur a coincident of post-partum hemmorrhage of the mother with the necessity of resuscitating of her new-born child.

If the time allotted to my paper were not limited I could illustrate this with many interesting cases from my private practice, but many of you, I am sure, have been handicapped under such trying circumstances more than once. Resuscitation of the new-born brings to my mind the possibility of a premature but viable child and the necessity of placing it in an incubator if we want to give it a fair chance of survival. Nobody will deny the danger of transportation of a new-born under the aforesaid circumstances. I can only mention here in a few words the great advantage of a hospital for women in case she should happen to develop a post-partum rise of temperature; a leucocyte count, examination of tears, systematic temperature, pulse and respiration charts and other means of correct clinical observation will lead, in many cases, to a diagnosis of sepsis or to a positive exclusion of this dreaded complication and consequently to rational treatment. While in our private practice in the homes of our patients the diagnosis and therapy are quite often of a haphazard character.

Let us consider now the proposition to send obstetrical cases to hospitals only when trouble is anticipated. I consider this erroneous. While there exists a very small number of obstetrical cases in which absolute indications for interference are present before labor begins, such cases are rare indeed. In the great majority of cases the indications are relative and sometimes appear quite suddenly, as for instance, the threatening death of the child. This indication is considered by some obstetricians of repute as the most frequent one in the choice of forceps operations. I mentioned already prolapse of the cord as an unexpected indication for obstetrical operation. Suppose again we have a face presentation—chin posteriorly—we certainly will wait for anterior rotation. After due allowance for nature we try to correct the position; let us say we fail. What are we to do? Wait longer and let the child die, or maybe expose the parturient to rupture of the uterus? Will we perforate a living child or shall we perform pubiotomy or Cesarean section in the house of the patient, or will we transport her at the eleventh hour to a hospital where she belonged from the start? That cases of placenta previa and of eclampsia belong to maternities, very few, who are familiar with their serious character, will deny. But nowadays such cases are mostly transported to materities only when they are moribund or after they have been in many cases previously infected in their homes.

Stroganoff, of Moscow, who has the lowest mortality of eclampsia on record, claims emphatically that not more than one per cent. of eclampsia patients should die, if they are treated rationally at the appearance of the first prodromata. An ophthalmoscopical examination may reveal neuro-retinitis and hemorrhage into the retina before any positive symptoms are present. A study of blood pressure may be of great value. It is stated that convulsions never occur in cases in which the blood pressure is not above 160. These measures may be of great help in differentiating the diagnosis between puerperal eclampsia, hysterical, or a typical epileptical convulsion. But such means, though easily applied in any well conducted hospital, are out of our reach in general practice except in families of the very rich.

It is impossible for me to go through each and every complication which is apt to occur during or after labor. But who will deny that the chances of mother and child are infinitely much better in an institution built and arranged for that purpose with the assistance of physicians and trained nurses than in a private house with everything defective as to perfect asepsis.

That women can be and often are safely delivered even under the most adverse surroundings has been proven by ages. That an ingenious and skillful man may convert kitchen utensils into operating tables and sterilisers, and kitchen mechanics into efficient assistants and still have good results, I certainly will not deny. In the first instances it equals "a happy guess of ignorance and a blind kindness of nature."

In the second proposition the skillful obstetrician might have still better results, especially as far as the percentage of living and healthy children is concerned. A good surgeon is just as competent to operate his cases in private houses as a good obstetrician. Still we seldom hear nowadays of a carcinomatous breast, for instance, being amputated in the patient's home, provided there is a hospital within easy reach.

So much for the greater safety of mothers and their children, safety not only of life but of subsequent perfect health. There remains one more factor which cannot be neglected. The hospital as a routine place of delivery would have a great educational influence upon the mothers. Many of them would learn to nurse their children and how to perform this function cleanly and regularly. Now a number of mothers stop nursing their babes on the most flimsy pretext, encouraged, sometimes even advised by so-called "nurses." They would see how the eyes, mouth and cord are handled properly; they would learn not to be afraid to give plenty of water to their babes; they would learn to discard that abomination, the breast pump, and the like.

But the women and the children will be benefitted not only directly but also indirectly. By being confined in hospitals they will be instrumental in helping to properly educate the future generation of physicians and nurses. It is really surprising that with all the criticism so liberally heaped upon the heads of the younger obstetricians nobody is asking himself how to remedy this evil. In a paper read before the Section of Obstetrics, Buffalo Academy of Medicine, December 28, 1909, by Dr. W. P. Manton, Chairman of the Obstetrical Section of the American Medical Association "The Aftermath of Childbirth," he insisted that no interne of any hospital should have his certificate signed unless he can prove that he served the same term in obstetrical as he is obliged to serve in the surgical and medical wards. Good! but what is the use of serving, if there is no material in the wards.

The same applies to hospital nurses. While in all other branches of applied medicine, physicians avail themselves of the assistance of trained nurses, while it is a general consensus of opinion among physicians that trained nurses are quite often of incalculable importance in our modern art of healing, in the private practice of mid-wifery this assistance is a rare occurrence. Most of us are compelled to work with so-called practical nurses, or to be sincere and call them by their proper name, we are assisted by quack nurses, most of them absolutely ignorant women, occasionally women with less than a smattering of the art of nursing. To ascertain the causes why trained nurses are in the majority of cases unwilling to take obstetrical cases, I interviewed quite a number of them. Their reasons can be classified under three categories. Most of the trained nurses mentioned as a reason the uncertainty of time of such engagements, sometimes several weeks. In waiting for an obstetrical case they are obliged to refuse other work. This is a straight financial argument. Other nurses claim that the obstetrical work is harder, inasmuch as they have two patients to take care of. This is a masked financial reason. Some others were sincere enough to admit that they do not feel properly qualified to do this kind of work as they did not see enough of this material during their training in school. This is a moral argument. You never hear this last argument from the so-called practical nurses and certainly will never expect to hear such a doubt from the fossils of obstetrics of former centuries, the midwives.

It is not within the scope of this paper to discuss the midwife problem. The fact is, however, that within the last three months in a single hospital in our city there died three women delivered by midwives in one single section, all of them after normal labors, every one of them sent to the hospital or, rather, dumped into the institution when practically moribund. This fact alone ought to be sufficient to make us consider not only the advisability but the crying necessity for most women to seek refuge in maternities. There certainly will die in Buffalo this, and the following years, more women and children during and after labor than from many communicable diseases or epidemics. Yet this dreadful state of affairs seems not to impress either the public or even our profession at large.

The medical profession, if it considers the problem carefully, will find that it has besides moral, also some selfish reasons favoring hospital treatment of parturient women. We hear so often reproaches directed against the general practitioner for their poor obstetrical work. We are apt to resent these as too harsh and injudicious. Although we must admit that there is much truth in this criticism, we feel that it is not the fault of each and everybody individually, but that there must be some broader underlying causes. The gynecologists who feed on our mistakes are the severest accusers. They claim that about 50 per cent. of their work is furnished by poor obstetrics. If we eliminate sepsis, which we must admit is less and less common in the hands of the younger generation of physicians, the most important factor in maiming women and their offspring is lack of indication and hastening labor by too early interference, or to apply Price's stigma "Junk Obstetrics." I do not intend to offer here an excuse for this kind of obstetrical work; it is certainly very deservedly condemned. The fact, however, that this practice is so general should make us look for its causes. It would not require much acumen to find that the principal reason here, as in most of our actions is an economic one.

The physician who, in the beginning of his practice takes an obstetrical engagement does so, either because he is in great financial distress and is satisfied to work sometimes for wages which would deprive a skilled workingman the membership in his union, or he takes a midwifery case as a stepping-stone for his family practice of the future. While he is young and has little experience and trusts more for success to his Kelley pad than to his textbooks, he is willing and can afford to spend sometimes days and nights with a single case. According as his practice increases, the doctor has to "make hay while the sun shines," he cannot miss his office work, and if a parturient does not get through in a certain hour, so much the worse for the parturient, the baby, the cervix, the perineum, sometimes the bladder and rectum. If the physician who did a hard day's work has to stay at his patient's bedside a whole night, you may be prepared to see the forceps about 7 A. M., so that he may be free and ready for another day's work. The physician certainly does not feel at ease to leave his patient undelivered. He is well aware that should the child be born during his absence he will be more severely criticised, even if no more damage has been done than if he would have a tear of the third degree while present on the case. Nay! in sewing up such a tear he may show his great operative skill and charge an extra fee, and if a glib talker, may even convince the gallery of the sick room that it was an absolute necessity to tear through the sphincter—that he saved the poor woman's life, and the like.

When the time comes, and come it must—that women will realise how much better chances they have in maternities than at home, how much safer they are under the care of properly trained nurses, the rôle of the attending obstetrician will radically change. He will diagnose the beginning of labor, make out the presentation, if possible the position, ascertain as to the condition of the child and will send his patient to the hospital. He will not need to hang around for many hours while the patient has slight pains with long intervals. He will be notified by the house physician when the character of the pains requires his presence, or if any complications set in. He will not need to worry that occasionally under such circumstances the baby may be born in his absence. His patient is not left to herself under the "care" of a fully or three-quarter ignorant "practical" nurse. There is a specially trained nurse, a young, but instructed house physician, who will know how to save the perineum, aseptically tie the cord, and the like. Even in a case of a post-partum hemorrhage he will not lose his head. The third stage of labor should not be tampered with. Most authorities agree today on non-interference as the best plan—but this stage may last an hour or more, and how many of you in a private house, after a woman has been delivered will wait a couple of hours for the placenta?

Nowadays in private practice the attending obstetrician, leaving a newly delivered woman, actually leaves her at her own risk. A secondary post partum hemorrhage, although exceptional, does occasionally occur. Who shall help a woman in a private house? She may (and sometimes does) bleed to death before the physician can be located and reach her bedside.

The time is ripe for the physicians to begin a campaign of education among their patients, to instruct them that the safest place for them to bear their children is a hospital. We insure ourselves against fire and accidents, we carry liability policies for ourselves and our automobiles. It is only just that we should see to it that our patients are appropriately insured against any and all possible accidents of parturition. From whatever point we consider this proposition we come to the same conclusion. In my own practice I notice that more and more women are coming to this conclusion. The number of patients who ask me unsolicited to find them accommodations in a hospital during their confinement is increasing slowly but gradually. Some of my friends relate to me the same experience in their practice. The increase in the number of parturients in hospitals certainly will be a great help in rounding up the education of young graduates who take interneships in hospitals, and most of them do so nowadays. The trained nurse will be only too glad to take care of obstetrical cases—in hospitals. And lastly, the physicians themselves will, under changed conditions, consider obstetrical work as less of a burden and of more real interest. They will be able to take care of their patients with full satisfaction because they will conduct labor according to strict indications. The only dissatisfied parties will be the class of midwives and practical nurses who will thus be eliminated and the "gossiper-physician" who will miss his audience of neighbors, grandmothers, and the like.

In choosing this subject for my paper I was well aware that I would not have smooth sailing. When I began to systematise my material I was considering whether I should not give up the task, but the subject has been occupying my mind so much during the last few years that it became my "hobby" and I wished to get rid of it—to deliver myself, so to say—of it. I hope this product will not be a still-born one. If it should appear to be premature I beg those of the younger members of our section who feel some sympathy for this idea to adopt it, put it in an incubator of patience and deliver it at the proper time.

415 Franklin Street.

